# Abrasions of the Teeth

**Published:** 1891-12

**Authors:** A. F. Townsend

**Affiliations:** Worcester, Mass.


### ABRASIONS OF THE TEETH.[1]

BY DR. A. F. TOWNSEND, WORCESTER, MASS.

Of the number of phases which this trouble with the teeth presents to the practitioner of dentistry, I shall refer to but one. The character of abrasion which I shall consider is the excessive wearing away of the grinding surfaces of the teeth by mastication. This form of abrasion is the most common, and for that reason calls for treatment more frequently than any other. Every practitioner has

---

[1] Read at Union Dental Meeting, Boston, October 29, 1890.

more or less experience with it, and while some cases are readily managed, others present a very difficult problem as to the best method to be adopted in their treatment.

In those where the abrasion is but slight and the surfaces of the teeth have the cupped appearance due to the more rapid wearing away of the dentine, I have usually resorted to filling the depressions flush with the surrounding enamel, and have found the results generally satisfactory where the wear in the patient's mouth is not rapid. In some cases I have found this exposed dentine acutely sensitive to touch, aggravated by any acid food, rendering the teeth anything but comfortable.

In treating these acutely sensitive surfaces I have given temporary relief by applying nitrate of silver. I use this agent by scraping into a powder a portion of the caustic from the stick or pencil. A small amount of the powdered caustic is then taken and applied directly to the sensitive surface, and secured there by covering it with a thin layer of cement, and the patient instructed to be careful for a few hours and not dislodge the stopping, at the end of which time they may scale it off themselves. This treatment leaves a blackened surface, and is objectionable where it may show. Sensitive abrasions treated in this manner give a considerable period of comfort. Where more permanent results are demanded, sufficient of the dentine is cut away to get depth enough for a gold filling, which I have sometimes found necessary to insulate by a very thin layer of gutta-percha or cement.

The cases I have so far mentioned are common and occur almost daily in practice.

In the model which I show you the teeth are worn very badly, the inferior incisors suffering the most. All the inferior incisors and cuspids have become worn until death of the pulp has occurred from exposure and have required treatment. The devitalization took place at intervals, if I remember correctly, of about a year.

The surfaces of the dentine are extremely sensitive, but not enough to cause much trouble in mastication. In 1881 or 1882 I had occasion to cap the inferior sixth-year molars and bicuspids, also the superior sixth-year molar on the left side. This was done chiefly on account of the sensitiveness of these particular teeth. This operation has arrested the wearing of the teeth filled, which antagonized only with those which were not filled in the upper jaw. This is shown very clearly in the model. The two lower molars and bicuspids were capped. These have become worn but slightly, while the wear in the corresponding teeth in the upper jaw, you will observe, has been excessive. The left superior and inferior sixth-year molars have worn about equally. The capping in the inferior molar came out a few months ago on account of the retaining groove on one side being obliterated by wear. The capping was literally worn out. The habit of gritting his teeth adds largely to the natural wear, if it is not the chief cause of this excessive destruction of the surfaces of these teeth. This man is, I think, about forty-five years of age, of a strong, sturdy physique, and will probably continue to grit his teeth as long as he has any, so any operation upon them must necessarily be of a very substantial character to possess any value to him.

For a number of years I have used gold-foil and built up the teeth, in cases like the model, one tooth after another, until I had, in my judgment, a sufficient thickness of gold to last. I think it frequently happens that in operations of this kind, where you desire the greatest durability, you are frequently disappointed. Where there is a great amount of abrasion, you may expect a correspondingly hard wear on the fillings or caps with which you protect the teeth.

One case which I capped in this manner in July, 1883, eighteen teeth were laboriously built up until the caps were a little less than one-eighth of an inch in thickness. I have just found it necessary to repeat this work, as a number of the caps had worn almost through. Four more teeth had to be attended to which were not capped at the time the operation was first done. These caps of pure foil have worn as well as gold could wear under the severe action which was brought to bear upon them. In restoring these caps I used Williams's gold and platina folds, and expect greater durability from its adoption. I have not used this material long enough to have any decided opinion upon its merits for hardness and durability.

My experience with a number of cases of like character had induced me to seek for some more permanent or durable material for this purpose than gold-foil. Amalgam can be used in some instances to advantage, but is not suitable for all conditions, particularly if the operation is to show.

The method employed for the last few years in capping teeth of this class I will attempt to describe. Beginning with a central incisor which is badly worn away, I proceed to grind the tooth level upon its articulating surface. The idea is to make the enamel on a level plane. The edge of enamel is then bevelled with a disk or file; I prefer a disk. Two holes are then drilled, usually one on each side of the vicinity of the pulp,—assuming that these teeth have live pulps,—to a depth of about one-tenth of an inch or deeper. I have found that depth sufficient for all purposes. I then make a ferrule of pure gold, which I fit accurately to the bevelled edge of the enamel by bevelling the inside edge of the ferrule. The gold should be a trifle larger than required, and only a trifle, as I wish this ferrule to form the surface of my cap, and if made too large it will have to be ground off in finishing. After the ferrule is fitted and made of sufficient height for the thickness of the cap, I take a sheet of No. 4 cohesive foil, which I fold to thirty-two thicknesses and anneal in the alcohol flame. This I lay over the end of the tooth and press it up firmly with my finger, the foil adapting itself very readily to the end of the tooth, with the edges projecting over. I then burnish the foil lightly to the edge. The ferrule is then taken and laid over this piece of foil and pressed firmly into position with the finger, and held there while the pins, which I have previously prepared, are thrust through the thin foil into the holes made in the teeth for them. A piece of beeswax is then taken, about large enough to fill the ferrule, warm it slightly in the hand and press it firmly into the ferrule. It is then removed from the tooth, the wax holding all the pieces in proper position. I then invest it, and after the investment has hardened I wash out the wax with hot water and flow into the ferrule eighteen- or twenty-carat gold solder until it is full. After removing from investment I try it upon the tooth, and with a corundum-stone and burnisher proceed to finish it. After it is roughly finished, I cement into place and then put on the final finish. I have been using this method for capping teeth where a durable cover is needed for some time, and have between thirty and forty caps now in service apparently as secure as ever. The labor of putting them on is not as tiresome for patient or operator as the method of capping with foil, and with a little practice they can be done much more rapidly. This method has not had the test of time which is necessary to prove success or failure, but so far I have every reason to hope for successful results. As eighteen-carat solder is more resisting than gold-foil in like proportion, I expect greater durability.

As the feelings and endurance of the patient are of some consideration in an extensive dental operation, as well as the tax upon nerve and muscle, the operator will find that he can accomplish more work of this character with comparative comfort to himself and patient if the method I have described be adopted.

